# Survival benefit of neoadjuvant hepatic arterial infusion chemotherapy followed by hepatectomy for hepatocellular carcinoma with portal vein tumor thrombus

**DOI:** 10.3389/fphar.2023.1223632

**Published:** 2023-09-19

**Authors:** Zili Hu, Zhenyun Yang, Jiongliang Wang, Yizhen Fu, Zhiwen Hu, Zhongguo Zhou, Minshan Chen, Yaojun Zhang

**Affiliations:** ^1^ Department of Liver Surgery, Sun Yat-sen University Cancer Center, Guangzhou, China; ^2^ Collaborative Innovation Center for Cancer Medicine, State Key Laboratory of Oncology in South China, Sun Yat-Sen University Cancer Center, Guangzhou, China

**Keywords:** HCC, hepatocellular carcinoma, PVTT, portal vein tumor thrombus, HAIC, neoadjuvant hepatic arterial infusion chemotherapy, hepatoectomy, neoadjuvant therapy

## Abstract

**Background/purpose:** The prognosis of hepatocellular carcinoma (HCC) patients with portal vein tumor thrombus (PVTT) is generally poor and hepatectomy is optional for these patients. This study aims to explore the survival benefits of neoadjuvant hepatic arterial infusion chemotherapy (HAIC) for resectable HCC with PVTT.

**Methods:** This retrospective study included 120 resectable HCC patients with PVTT who underwent hepatectomy, from January 2017 to January 2021 at Sun Yat-sen University Cancer Center. Of these patients, the overall survival (OS) and recurrence-free survival (RFS) of 55 patients who received hepatectomy alone (Surgery group) and 65 patients who received neoadjuvant HAIC followed by hepatectomy (HAIC-Surgery group) were compared. Logistic regression analysis was conducted to develop a model predicting the response to neoadjuvant HAIC.

**Results:** The OS rates for the HAIC-Surgery group at 1, 3, and 5 years were 94.9%, 78%, and 66.4%, respectively, compared with 84.6%, 47.6%, and 37.2% in the Surgery group (*p* < 0.001). The RFS rates were 88.7%, 56.2%, and 38.6% *versus* 84.9%, 38.3%, and 22.6% (*p* = 0.002). The subgroup analysis revealed that the survival benefit of neoadjuvant HAIC was limited to patients who responded to it. The logistic model, consisting of AFP and CRP, that predicted the response to neoadjuvant HAIC performed well, with an area under the ROC curve (AUC) of 0.756.

**Conclusion:** Neoadjuvant HAIC followed by hepatectomy is associated with a longer survival outcome than hepatectomy alone for HCC patients with PVTT and the survival benefit is limited to patients who respond to neoadjuvant FOLFOX-HAIC.

## 1 Introduction

Hepatocellular carcinoma (HCC) is the seventh most frequent cancer and the third highest leading cause of cancer-related mortality globally (Sung et al., 2021). The portal venous system may be invaded by hepatoma cells, resulting in portal vein tumor thrombus (PVTT). It was previously reported that PVTT was identified in 10%–40% of patients with HCC at the time of initial diagnosis (Llovet et al., 1999; Minagawa and Makuuchi, 2006).

The prognosis of patients with PVTT is generally poor with a median survival time (MST) of 2–4 months with best-supportive care (Llovet et al., 1999; Schöniger-Hekele et al., 2001; Minagawa and Makuuchi, 2006), 10.7 months with sorafenib (Llovet et al., 2008), 7–10 months with transarterial chemoembolization (TACE) (Chung et al., 2011; Luo et al., 2011), 6.5–14 months with hepatic arterial infusion chemotherapy (HAIC) (Eun et al., 2009; Kim et al., 2010; He et al., 2017), 9.6–10.9 months with external beam radiation therapy (RT) (Toya et al., 2007; Nakazawa et al., 2014) and 6–16.9 months with transarterial radioembolization (TARE) (Kulik et al., 2008; Salem et al., 2010; Sangro et al., 2011; Memon et al., 2013). Recently some studies have reported that patients with PVTT can benefit from surgical resection, which is the only treatment that may offer these patients a chance for long-term survival (MST, 21.2–25.4 months) (Tanaka et al., 1996; Poon et al., 2003; Kokudo et al., 2016). Even so, the survival of patients with PVTT is dissatisfactory. Neoadjuvant therapy has been advocated to improve the postoperative prognoses of these patients. Neoadjuvant treatment including sorafenib and/or radiotherapy together with hepatectomy was proven to prolong the survival of these patients (Irtan et al., 2011; Kermiche-Rahali et al., 2013).

Regarding HAIC, there have been attempts to develop various regimens, including cisplatin-based regimens (CDDP: low dose cisplatin, FP: cisplatin, 5-fluorouracil) (Yamashita et al., 2011; Ikeda et al., 2013) and oxaliplatin-based regimens (FOLFOX: oxaliplatin, fluorouracil, and folinic acid) (He et al., 2017; Lyu et al., 2018; He et al., 2019; Li et al., 2022) mainly. Recently, FOLFOX-HAIC showed promising efficacy in treating HCC with PVTT. Li’s research revealed that FOLFOX-HAIC yielded significantly better treatment responses than TACE for patients with unresectable large HCC (Li et al., 2022), and the combination of FOLFOX-HAIC and sorafenib yielded significantly better treatment responses than sorafenib alone for advanced HCC patients with PVTT; 16 (12.8%) patients received curative hepatectomy thereafter in the combination therapy group but 1 (0.8%) patient in the sorafenib group (He et al., 2019). HAIC is recommended as the standard treatment for hepatocellular carcinoma with portal vein tumor thrombus by Japanese guidelines. However, the benefit of neoadjuvant FOLFOX-HAIC for resectable HCC with PVTT has not been reported previously.

In this study, we aimed to explore the survival benefit of neoadjuvant FOLFOX-HAIC for resectable HCC with PVTT and filtrate proper candidates to accept neoadjuvant FOLFOX-HAIC.

## 2 Methods

### 2.1 Patients

**FIGURE 1:**
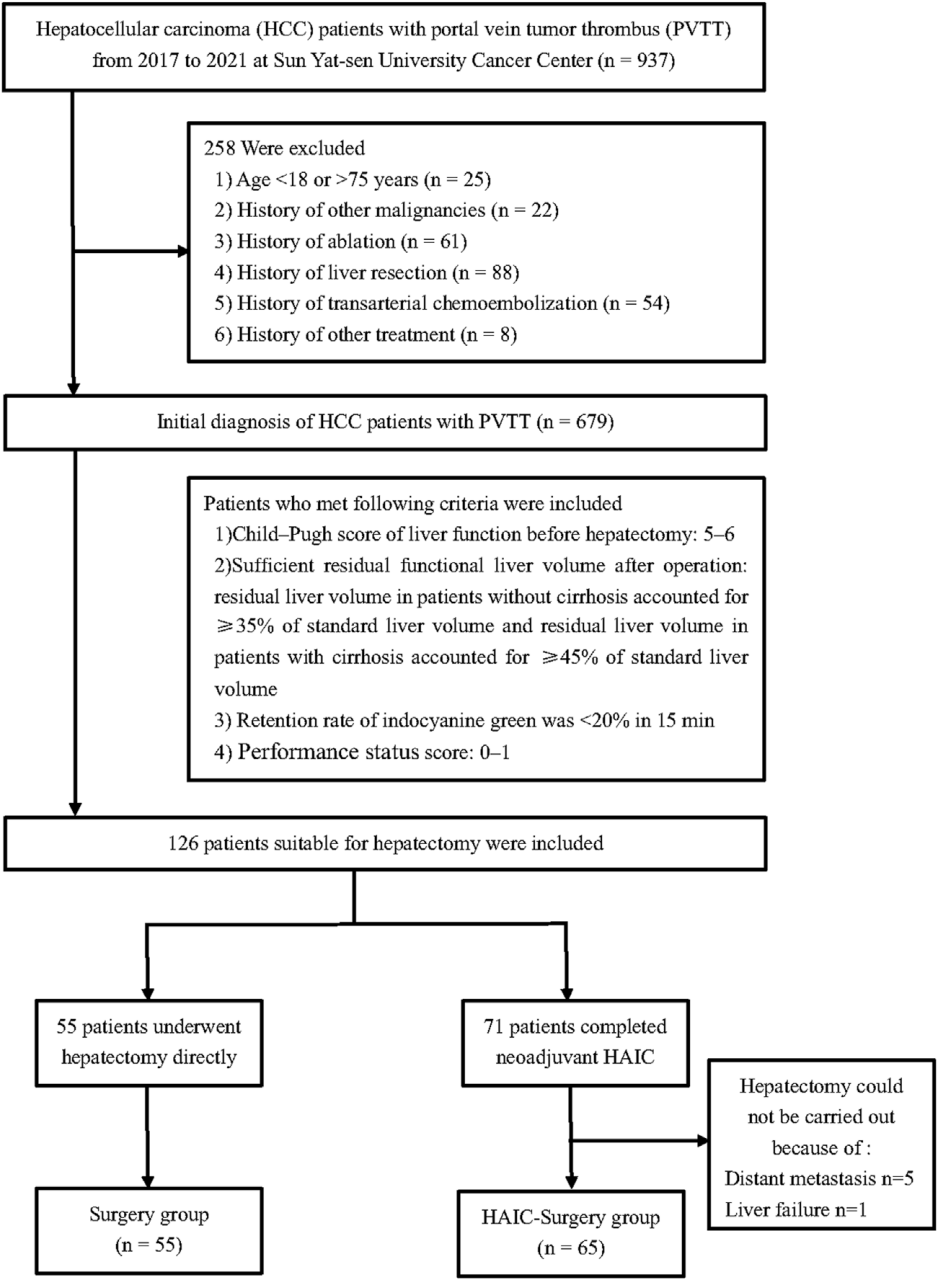
Flow diagram for the patient selection process.

From January 2017 and January 2021, all patients between 18 and 75 years old who had initial diagnosis of HCC with PVTT at Sun Yat-sen University Cancer Center (SYSUCC) were included into this study. Patients with history of other malignancies and history of ablation, liver resection, TACE or other treatment were excluded. The indications to apply hepatectomy were as follows: 1) Child–Pugh score of liver function before hepatectomy: 5–6; 2) Sufficient residual functional liver volume after operation: residual liver volume in patients without cirrhosis accounted for ≥35% of standard liver volume and residual liver volume in patients with cirrhosis accounted for ≥45% of standard liver volume; 3) Retention rate of indocyanine green was <20% in 15 min; 4) Eastern Oncology Cooperative group score: 0–1. After screening, a total of 126 patients were deemed appropriate for hepatectomy. Within these, 55 patients underwent hepatectomy directly, while 71 patients received neoadjuvant HAIC. However, 5 of the patients who received neoadjuvant HAIC were found to have distant metastasis and were unable to undergo hepatectomy, and 1 patient experienced liver failure and was also unable to undergo the procedure (Figure 1). This study was conducted according to the ethical guidelines of the 1975 Declaration of Helsinki. This research was approved by the institutional review board of Sun Yat-sen University Cancer Center.

### 2.2 HAIC procedures

HAIC was performed every 3 weeks. In every cycle of treatment, femoral artery puncture and catheterization were performed on day 1. The following regimen was perfused via the hepatic artery: oxaliplatin, 85 mg/m^2^, from hour 0 to 2 on day 1; leucovorin, 400 mg/m^2^, from hour 2 to 3 on day 1; fluorouracil, 400 mg/m^2^, bolus at hour 3; and 2,400 mg/m^2^ over 46 h on days 1 and 2. The catheter and sheath were removed immediately after HAIC was completed. Repetitive catheterization was performed in the next HAIC cycle. Magnetic resonance imaging (MRI) was performed every 6 weeks during neoadjuvant HAIC and efficacy was estimated according to modified Response Evaluation Criteria in Solid Tumors (mRECIST) (Lencioni and Llovet, 2010). After estimating the treatment response, hepatectomy was offered to patients when curative resection was determined, i.e., all tumors including PVTT, were achievable with sufficient hepatic functional reserve. The treatment was stopped when there was progressive disease, development of extrahepatic diseases or evidence of liver failure. Although patients were estimated as progressive disease (PD), hepatectomy was conducted if curative resection was achievable.

### 2.3 Follow-up

After surgery, all patients were observed in the first month, every 3 months within 2 years and then every 6 months thereafter. Laboratory tests (including serum AFP level, liver function tests, and blood tests) and MRI were conducted in follow-up examinations. If recurrence occurred, recurrent HCC was treated by further surgical resection, radiofrequency ablation, interventional therapy or targeted drug therapy according to the tumor recurrence status and the patient’s liver function.

### 2.4 Outcomes and definitions

The primary end point was overall survival (OS), defined as the interval between hepatectomy and death from any cause or the date of the last follow-up. The secondary end point was recurrence-free survival (RFS), defined as the time from the date of hepatectomy to the date at which HCC recurred. The overall response rate (ORR) is defined as the sum of complete response (CR) and partial response (PR) rates. Cirrhosis was defined histologically according to the pathology of resected liver specimens. PVTT was categorized into third-order branch (Vp1), second-order branch (Vp2), first-order branch (Vp3) and main trunk/contralateral branch (Vp4) (Kudo et al., 2011) based on the radiological findings (Supplementary Figure S1A). Histologic grade of tumor differentiation are based on the Edmondson–Steiner (ES) classification (Edmondson and Steiner, 1954): ES stage I, II, III, IV.

### 2.5 Statistical analysis

All statistical analyses were with R 3.63 (R Foundation for Statistical Computing, Vienna, Austria, https://www.R-project.org/) and SAS (version 26.0, SAS Institute, Cary, NC). Categorical variables were presented as frequencies and percentages and were compared by the chi-square test between two groups. Continuous variables were described as the mean ± standard deviation and median with interquartile range for parametric and nonparametric variables, respectively, and were compared by Student’s *t*-test or nonparametric test. Propensity score matching (PSM) was conducted using the “MatchIt” R package, with a caliper width set to 0.2 of the standard deviation of the logit of the propensity score. Survival curves were performed by Kaplan-Meier method and compared by log-rank test. Univariable and multivariable Cox proportional hazards models were performed to assess the risk factors for recurrence and overall survival. The area under the ROC curve (AUC) was measured to evaluate the predictive value of the logistic model.

## 3 Results

### 3.1 Baseline characteristics of the patients

This study consecutively collected 120 HCC patients with PVTT who underwent hepatectomy. Among them, 55 (45.8%) patients received surgery alone (Surgery group), and 65 (54.2%) patients were treated with neoadjuvant FOLFOX-HAIC followed by surgery (HAIC-Surgery group). The average times of FOLFOX-HAIC procedures was 3.2. Their average age was 50.3 [95% confidence interval (CI), 39.7–60.9] years. 107 (89.2%) patients were male. The average tumor size was 9.38 (95% CI, 5.57–13.19) cm. A total of 109 (90.8%) patients were infected with HBV and 74 (61.7%) patients were confirmed to have cirrhosis.

The median follow time was 34.8 (95% CI, 29.2–40.4) months in all patients, 38.7 (95% CI, 27.7–49.8) months in Surgery group and 33.8 (95% CI, 28.3–39.2) months in the HAIC-Surgery group (*p* = 0.346). Compared with the Surgery group, the HAIC-Surgery group had significantly more patients with longer PT (12.34 s vs. 11.92 s, *p* = 0.01) and more patients with Vp3/4 of PVTT (67.7% vs. 45.5%, *p* = 0.023). The other characteristics were not significantly different between the two groups (Table 1). PSM (1:1 matching) according to PT and extent of PVTT analysis generated a cohort of 43 and 43 patients in the Surgery and the HAIC-Surgery groups, respectively. The characteristics of the two groups were balanced (Table 1). 58 patients had tumor recurrence in the entire cohort and 41 patients in the PSM cohort. In the entire cohort, 6 (23.1%) patients received radical treatment (re-resection or ablation) in the Surgery and 9 (28.1%) patients in the HAIC-surgery group (*p* = 0.892). In the PSM cohort, 6 (25%) patients received radical treatment in the Surgery and 7 (41.2%) patients in the HAIC-surgery group (*p* = 0.450) (Supplementary Table S1).

**TABLE 1:** Baseline characteristics of patients in entire cohort and PSM cohort.

	Entire cohort			PSM cohort		
	Surgery group	HAIC-Surgery group	*p*-value	Surgery group	HAIC-Surgery group	*p*-value
	(*n* = 55)	(*n* = 65)		*(n* = 43)	(*n* = 43)	
Age (years)	51.2 ± 10.3	49.5 ± 10.8	0.409	50.0 ± 10.0	47.4 ± 10.9	0.259
Gender (N, %)			1			0.737
man	49 (89.1)	58 (89.2)		39 (90.7)	37 (80.6)	
woman	6 (10.9)	7 (10.8)		4 (9.3)	6 (14.0)	
PS score			0.979			1
0	50	59		40	3950	
1	5	6		3	4	
HBV infection (N, %)			0.731			0.265
absence	4 (7.3)	7 (10.8)		2 (4.7)	6 (14.0)	
presence	51 (96.7)	58 (89.2)		41 (95.3)	37 (86.0)	
HCV infection (N, %)			0.458			1
absence	54 (98.2)	65 (100)		42 (97.7)	43 (100)	
presence	1 (1.8)	0 (0)		1 (2.3)	0 (0)	
Complications of surgery (N, %)			0.155			0.093
absence	44 (80)	59 (90.8)		35 (81.4)	41 (95.3)	
presence	11 (20)	6 (9.2)		8 (18.6)	2 (4.7)	
Cirrhosis (N, %)			0.551			0.657
absence	19 (34.5)	27 (41.5)		15 (34.9)	18 (41.9)	
presence	36 (65.5)	38 (58.5)		28 (65.1)	25 (58.1)	
Tumor size (cm)	9.17 ± 4.28	9.50 ± 3.39	0.663	9.60 ± 3.91	9.70 ± 3.66	0.901
Tumor number (N, %)			0.974			0.818
solitary	37 (67.3)	45 (69.2)		28 (65.1)	30 (69.8)	
multiple	18 (32.7)	20 (30.8)		15 (34.9)	13 (30.2)	
Differentiation (N, %)			0.151			0.384
I, II	19 (34.5)	32 (49.2)		16 (37.2)	21 (48.8)	
III, IV	36 (65.5)	33 (50.8)		27 (62.8)	22 (51.2)	
Resection margin, cm	1 (0.2, 1.75)	1 (0.1, 1.5)	0.269	1 (0.2, 1.5)	1 (0.1, 1.5)	0.532
Platelet (N, %)			0.458			1
>100 x10^3^/mm^3^	54 (98.2)	65 (100)		42 (97.7)	43 (100)	
≤100 x10^3^/mm^3^	1 (1.8)	0 (0)		1 (2.3)	0 (0)	
PT(s)	11.92 ± 0.78	12.34 ± 0.98	0.01	11.96 ± 0.80	12.07 ± 0.82	0.504
Albumin (g/dL)	43.21 ± 3.30	42.84 ± 3.56	0.558	42.89 ± 3.40	43.6 ± 2.9	0.297
Total bilirubin (mg/dL)	12.7 (9.75, 18.1)	14.6 (10.45, 18.18)	0.378	12.7 (10.1, 18.1)	14 (10.4, 18)	0.378
ALT (U/L)	42.2 (29.25, 61.8)	46.15 (33.15, 69.95)	0.209	43 (35.12, 64.9)	46.5 (34, 65.25)	0.776
AST (U/L)	45.4 (35.85, 69.2)	50.3 (38.80, 75.6)	0.296	50.1 (39.95, 70.85)	50.2 (39.35, 75.7)	0.928
AFP (N, %)			0.963			0.828
<400 ng/mL	24 (43.6)	27 (41.5)		18 (43.6)	20 (41.5)	
≥400 ng/mL	31 (56.4)	38 (58.5)		25 (56.4)	23 (58.5)	
ALBI (N, %)			0.107			0.547
Grade 1	50 (90.9)	51 (78.5)		38 (90.9)	35 (78.5)	
Grade 2	5 (9.1)	14 (21.5)		5 (9.1)	8 (21.5)	
Extent of PVTT			0.023			1
VP1/2	30 (54.5)	21 (32.3)		20 (46.5)	20 (46.5)	
Vp3/4	25 (45.5)	44 (67.7)		23 (53.5)	23 (53.5)	

Categorical variables are described as frequencies and percentages. Continuous variables are described as mean ± standard deviation and median with interquartile range for parametric and non-parametric variables, respectively. HBV: hepatitis B virus; HCV: hepatitis C virus; AFP, alpha fetoprotein; ALT, alanine aminotransferase; AST, aspartate aminotransferase; PT, prothrombin time; ALB, albumin; TBIL, total bilirubin; ALBI, Albumin-Bilirubin; PVTT, portal vein tumor thrombus.

### 3.2 Survival benefit of neoadjuvant FOLFOX-HAIC followed by hepatectomy

**FIGURE 2:**
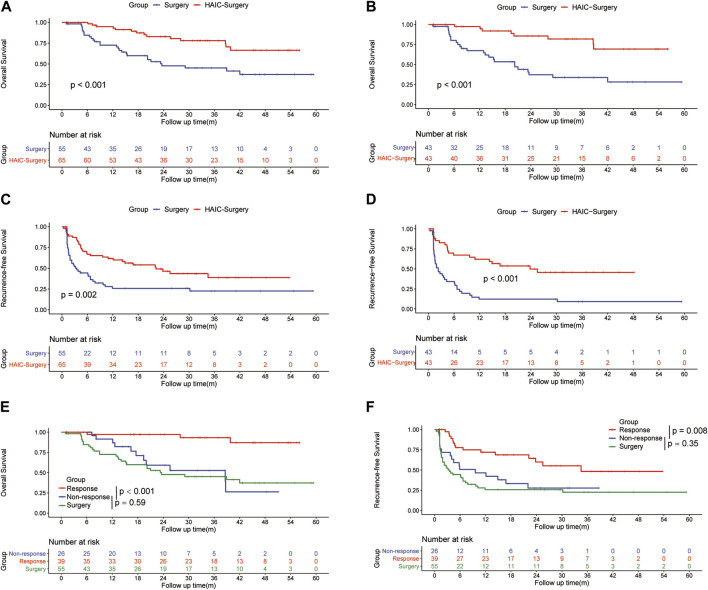
Kaplan–Meier curves of overall survival between the Surgery group and the HAIC-Surgery group in the entire cohort **(A)** and the PSM cohort **(B)**; Kaplan–Meier curves of HCC recurrence between the Surgery group and the HAIC-Surgery group in the entire cohort **(C)** and the PSM cohort **(D)**; Kaplan–Meier curves of overall survival **(E)** and HCC recurrence **(F)** among the Surgery group, Response group and Non-Response group in all patients.

In the entire cohort, the 1-, 3-, and 5-years OS rates were 84.6%, 47.6% and 37.2% in the Surgery group and 94.9%, 78% and 66.4% in the HAIC-Surgery, respectively (Figure 2A). In the PSM cohort, 1-, 3-, and 5-years OS rates were 67.3%, 33.9% and 28.2% in the Surgery group and 97.5%, 81.9% and 69.3% in the HAIC-Surgery, respectively (Figure 2B). The Neoadjuvant FOLFOX-HAIC showed a significant survival benefit for patients with PVTT (*p* < 0.001, entire cohort; *p* < 0.001, PSM cohort). When the 6 patients who were unable to undergo hepatectomy are included in the HAIC-Surgery group, patients in the HAIC-Surgery group still showed significant longer survival than the Surgery group (*p* < 0.001, Supplementary Figure S1B). A multivariate Cox regression analysis was performed and identified neoadjuvant FOLFOX-HAIC as a significant protective factor for survival (HR 0.310; 95% CI 0.716–0.637; *p* < 0.001). In addition, tumor size was identified as a significant factor associated with survival (Table 2).

**TABLE 2:** Univariate and multivariate analysis of risk factors for overall survival and recurrence-free survival.

Variables	Overall survival							Recurrence-free survival							
	Univariate analysis			Multivariate analysis				Univariate analysis				Multivariate analysis			
	HR	95% CI	P	HR		95% CI	*p*-Value	HR	95% CI	P		HR	95% CI	*P*	
Age (years)	1.003	(0.976–1.032)	0.158					0.995	(0.974–1.017)	0.675					
Sex (female: male)	0.428	(0.132–1.390)	0.158					0.688	(0.314–1.504)	0.348					
Tumor size (cm)	1.097	(1.016–1.177)	0.017	1.099		(1.028–10174)	0.006	1.087	(1.026–1.152)	0.005		1.084	(1.023–1.149)	0.006	
Tumor number (solitary: multiple)	1.916	(1.045–3.513)	0.036					1.810	(1.125–2.912)	0.014		1.661	(1.009–2.735)	0.046	
Tumor differentiation (high, medium: low)	1.573	(0.985–2.512)	0.058					1.665	(1.184–2.341)	0.003					
Cirrhosis (no: yes)	0.725	(0.394–1.335)	0.302					0.848	(0.528–1.362)	0.495					
Resection margin (cm)	0.898	(0.636–1.268)	0.541					0.741	(0.554–0.991)	0.043		0.676	(0.496–0.923)	0.014	
HBV infection (no: yes)	2.288	(0.553–9.469)	0.253					1.100	(0.476–2.54)	0.824					
Platelet, ×109/L (≥100:<100) (≥100:<100)	4.887	(0.656–36.433)	0.122					3.449	(0.47–25.333)	0.224					
AFP, ng/mL (<400: ≥400)	0.920	(0.498–1.698)	0.789					1.097	(0.684–1.758)	0.701					
ALT, U/L (≤50:>50)	0.674	(0.357–1.27)	0.222					0.784	(0.486–1.263)	0.317					
AST, U/L (≤40:>40)	1.149	(0.588–2.247)	0.684					1.488	(0.871–2.543)	0.145					
ALB, g/L (≥35:<35)	0.960	(0.879–1.049)	0.373					0.981	(0.921–1.044)	0.545					
TBIL, μmol/L (≤17.1:>17.1)	1.119	(0.589–2.127)	0.732					1.057	(0.642–1.738)	0.828					
PT, s (≤13.5; >13.5)	1.624	(0.638–4.136)	0.309					1.412	(0.645–3.091)	0.388					
ALBI grade (I: II)	1.332	(0.590–3.010)	0.490					1.316	(0.72–2.407)	0.372					
Extent of PVTT (Vp1/2; Vp3/4)	0.815	(0.512–1.297)	0.389					0.830	(0.586–1.175)	0.293					
Neoadjuvant HAIC (no: yes)	0.335	(0.176–0.637)	<0.001	0.310		(0.716–0.637)	<0.001	0.470	(0.294–0.752)	0.002		0.368	(0.228–0.596)	<0.001	

HR, hazard rate; CI, confidence interval; HBV, hepatitis B virus; AFP, alpha fetoprotein; ALT, alanine aminotransferase; AST, aspartate aminotransferase; PT, prothrombin time; ALB, albumin; TBIL, total bilirubin; ALBI, Albumin-Bilirubin; PVTT, portal vein tumor thrombus; HAIC, hepatic arterial infusion chemotherapy.

In the entire cohort, the 1-, 3-, and 5-years RFS rates of HCC were 84.9%, 38.3% and 22.6% in the Surgery group and 88.7%, 56.2% and 38.6% in the HAIC-Surgery, respectively (Figure 2C). In the PSM cohort, the 1-, 3-, and 5-years RFS rates of HCC were 12.2%, 9.2% and 9.2% in the Surgery group and 62.1%, 45.7% and 45.7% in the HAIC-Surgery, respectively (Figure 2D). The HAIC-Surgery group showed significantly lower recurrence rates than the Surgery group (*p* = 0.002, entire cohort; *p* < 0.001, PSM cohort). A multivariate Cox regression analysis was performed and also identified neoadjuvant FOLFOX-HAIC as a significant protective factor for recurrence (HR 0.368; 95% CI 0.228–0.596; *p* < 0.001). In addition, tumor size, tumor number and resection margin were identified as significant factors associated with recurrence (Table 2).

### 3.3 Subgroup analysis

The ORR of FOLFOX-HAIC was 60.0% (39 of 65 patients), 7 (10.8%) patients had CR, 32 (49.2%) had PR, 24 (36.9%) had stable disease (SD) and 2 (3.1%) had PD, estimated according to mRECIST (Lencioni and Llovet, 2010). Patients with CR and PR were defined as the Response group, patients with SD and PD were defined as the Non-response group. The 1-, 3- and 5-years OS rates were 97.1%, 87.0% and 87.0%, respectively, in the Response group *versus* 76.6%, 26.3% and 26.3% in the Non-response group (*p* < 0.0001, Figure 2E). The 1-, 3- and 5-years RFS rates were 77.8%, 48.3% and 48.3%, respectively, in the Response group *versus* 59.1%, 27.7% and 27.7% in the Non-response group (*p* = 0.008, Figure 2F). However, there was no difference in RFS (12.1 months vs. 3.5 months, *p* = 0.35) or OS (38.6 months vs. 23.6 months, *p* = 0.59) between the Non-response group and the Surgery group (Figures 2E, F). In addition, the number of HAIC producers did not influence the survival of patients in HAIC-surgery group (Supplementary Figures 1C, D).

**FIGURE 3:**
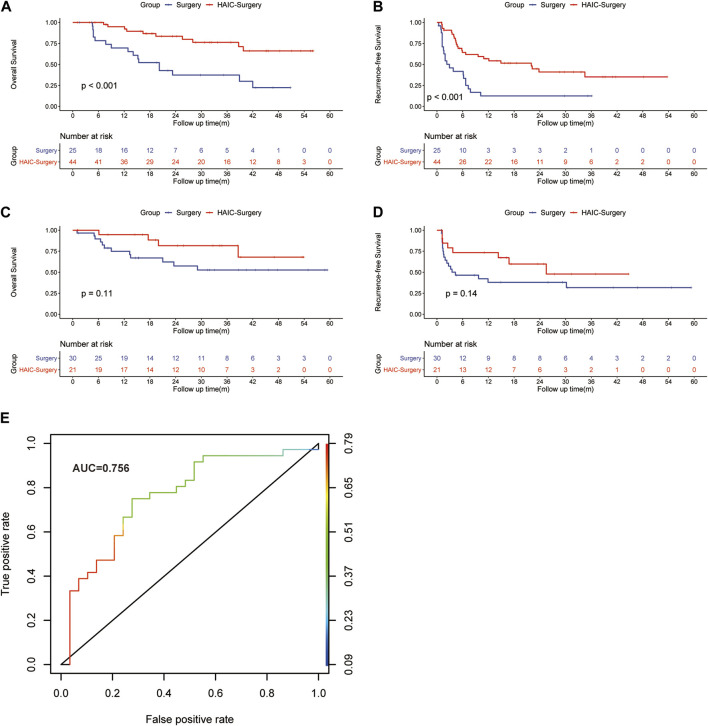
Kaplan–Meier curves of HCC overall survival **(A)** and recurrence **(B)** between the Surgery group and HAIC-Surgery group in patients with Vp3/4 of PVTT; Kaplan–Meier curves of overall survival **(C)** and HCC recurrence **(D)** between the Surgery group and HAIC-Surgery group in patients with Vp1/2 of PVTT. The ROC curve for the logistic model in predicting the response to neoadjuvant FOLFOX-HAIC **(E)**.

In patients with Vp3/4 of PVTT, the 1-, 3-, and 5-years OS rates were 69.6%, 37.4% and 22.4% in the Surgery group and 94.9%, 76.2% and 66.1% in the HAIC-Surgery, respectively (Figure 3A, *p* < 0.001). The 1-, 3-, and 5-years RFS rates of HCC were 22.5%, 22.5% and 22.5% in the Surgery group and 56.8%, 35.1% and 35.1% in the HAIC-Surgery, respectively (Figure 3B, *p* < 0.001). In patients with Vp1/2 of PVTT, the 1-, 3-, and 5-years OS rates were 74.9%, 52.7% and 52.7% in the Surgery group and 94.7%, 81.6% and 68.0% in the HAIC-Surgery, respectively (Figure 3C, *p* = 0.11). The 1-, 3-, and 5-years RFS rates of HCC were 64.4%, 31.7% and 31.7% in the Surgery group and 73.2%, 47.7% and 47.7% in the HAIC-Surgery, respectively (Figure 3D, *p* = 0.14). Neoadjuvant FOLFOX-HAIC showed a recurrence and survival benefits in patients with Vp3/4 of PVTT. However, the recurrence and survival benefit of neoadjuvant FOLFOX-HAIC was not significant in patients with Vp1/2 of PVTT. Patients with Vp3/4 of PVTT are more fit to accept neoadjuvant HAIC.

### 3.4 Safety

During HAIC procedure, the most common adverse events (AEs) about HIAC were elevated ALT (71.4%) and AST (72.9%), the levels of ALT and AST showed a significant increase on the day immediately following the completion of HAIC treatment, but then returned to normal within 1 week. The most common somatosensory AE was abdominal pain (40.0%), the routine administration of anisodamine and nonsteroidal anti-inflammatory drugs was implemented to prevent abdominal pain. Some patients suffered from leukopenia (34.4%), anemia (38.6%) and thrombocytopenia (32.9%), possibly due to the myelosuppression of fluorouracil. Details were shown in Table 3.

**TABLE 3:** Treatment-related adverse events about HAIC.

	Grades 1 and 2	Grades 3 and 4	Any grades
	No. (%)	No. (%)	No. (%)
Fever	9 (12.8)	0	9 (12.8)
Abdominal pain	28 (40.0)	0	28 (40.0)
Vomiting	15 (21.4)	1 (1.4)	16 (22.8)
Nausea	17 (24.3)	0	17 (24.3)
Diarrhea	6 (8.6)	0	6 (8.6)
Leukopenia	22 (31.4)	3 (4.3)	24 (34.3)
Anemia	27 (38.6)	2 (2.9)	27 (38.6)
Thrombocytopenia	19 (27.1)	6 (8.6)	23 (32.9)
Hyperbilirubinemia	15 (21.4)	0	15 (21.4)
Hypoalbuminemia	20 (28.6)	1 (1.4)	21 (30.0)
Elevated ALT	45 (64.3)	8 (11.4)	50 (71.4)
Elevated AST	44 (62.9)	9 (12.9)	51 (72.9)
Elevated creatinine	6 (8.6)	0	6 (8.6)

Abbreviations: ALT, alanine aminotransferase; AST, aspartate aminotransferase.

During surgery procedure, compared with the Surgery group, the HAIC-Surgery group had more patients with shorter postoperative hospital stays (8 days vs. 11 days, *p* < 0.001). Operation time, operative blood loss, operative blood transfusion, postoperative complications and 90-day mortality were not significantly different between the two groups, as shown in Table 4.

**TABLE 4:** Surgical features and short-term outcome between two groups.

	Surgery group	HAIC-surgery group	*p*-value
	(*n* = 55)	(*n* = 65)	
Postoperative hospital stays (days)	11 (9, 13.5)	8 (7, 12)	<0.001
Operation time (min)	180 (147.5, 210.5)	180 (150, 200)	0.973
Operative blood loss (ml)	300 (200, 500)	300 (200, 500)	0.325
Operative blood transfusion (N, %)			0.473
No	46 (83.6)	51 (78.5)	
Yes	9 (16.3)	14 (21.5)	
Postoperative complications (N, %)			0.202
Absent	44 (80)	59 (90.7)	
hepatic insufficiency	5 (9.1)	2 (3.1)	
bile leakage	1 (1.8)	2 (3.1)	
thorax/peritoneal effusion	3 (5.5)	1 (1.5)	
pulmonary/peritoneal infection	2 (3.6)	1 (1.5)	
postoperative hemorrhage	0 (0)	1 (1.5)	
intestinal obstruction	1 (1.8)	0 (0)	
90-day mortality (N, %)	3 (5.5)	3 (4.6)	1

Categorical variables are described as frequencies and percentages. Continuous variables are described as mean ± standard deviation and median with interquartile range for parametric and non-parametric variables, respectively.

### 3.5 The logistic regression model in predicting the response to neoadjuvant FOLFOX-HAIC

To select patients who can benefit from neoadjuvant FOLFOX-HAIC, we developed a logistic regression model. Univariate analysis identified serum AFP (*p* = 0.039) and CRP (*p* = 0.028) levels as independent factors (Table 5),which were used in the multivariate model. Higher AFP and lower CRP level were associated with the response to neoadjuvant FOLFOX HAIC. The final logistic regression model for predicting the response to neoadjuvant FOLFOX-HAIC within 3 months:

**TABLE 5:** Baseline characteristics of patients in HAIC-Surgery group.

	Non-response group	Response group	*p*-value
	(n = 26)	(n = 39)	
Age (years)	50.77 ± 10.94	48.72 ± 10.82	0.459
Gender (N, %)			0.693
Women	24 (92.3)	34 (87.2)	
Men	2 (7.7)	5 (12.8)	
HBV infection (N, %)			0.424
Absence	4 (15.4)	3 (7.7)	
Presence	22 (84.6)	36 (92.3)	
HCV infection (N, %)			1
Absence	26 (100)	39 (100)	
Presence	0 (0)	0 (0)	
Cirrhosis (N, %)			0.165
Absence	14 (54)	13 (33)	
Presence	12 (46)	26 (67)	
Tumor size (cm)	9.52 ± 3.69	9.49 ± 3.23	0.973
Tumor number (N, %)			0.411
Solitary	20 (76.9)	25 (64.1)	
Multiple	6 (23.1)	14 (35.9)	
Differentiation (N, %)			0.51
I, II	11 (42.3)	21 (53.8)	
III, IV	15 (57.7)	18 (46.2)	
Resection margin, cm	0.85 (0.02, 1.5)	1 (0.15, 1.5)	0.946
Platelet (x10^3^/mm^3^)	280 (210.5, 340.25)	233 (176.5, 301.5)	0.077
PT(s)	12.38 ± 0.98	12.32 ± 0.99	0.804
ALB (g/dL)	43.55 (40.92, 45.77)	43.3 (41.2, 45.13)	0.984
TBIL (mg/dL)	12.6 (8.93, 16.73)	15.15 (11.62, 19.9)	0.107
ALT (U/L)	46.25 (27.6, 65.03)	46.15 (35.35, 76.4)	0.367
AST (U/L)	50.1 (37.27, 65.83)	58.4 (40.15, 84.35)	0.335
AFP (ng/mL)	149.55 (19.69, 11315.75)	5767 (74.75, 53196)	0.039
CRP (mg/mL)	26.61 (6.06, 37.49)	7.96 (2.8, 16.11)	0.028
Extent of PVTT			0.33
VP1/2	10 (38.5)	11 (28.2)	
Vp3/4	16 (60.5)	28 (69.8)	
HAIC times			0.557
<4	14 (53.8)	21 (53.8)	
≥4	12 (46.2)	18 (46.2)	

Categorical variables are described as frequencies and percentages. Continuous variables are described as mean ± standard deviation and median with interquartile range for parametric and non-parametric variables, respectively. HBV: hepatitis B virus; AFP, alpha fetoprotein; ALT, alanine aminotransferase; AST, aspartate aminotransferase; PT, prothrombin time; ALB, albumin; TBIL, total bilirubin; ALBI, Albumin-Bilirubin; CRP, C-reactive protein; PVTT, portal vein tumor thrombus; HAIC, hepatic arterial infusion chemotherapy.

Logit (P) = -0.05 + 1.34(AFP)-0.015(CRP), the performance of the model was good with AUC of 0.756 (95% IC, 0.627–0.868; Figure 3E). The estimated probability at sensitivity and specificity maximum sum in predicting the response to neoadjuvant FOLFOX-HAIC are at a cut-off probability of 0.468, which means if the estimated probability was <0.468, it was classified into the Non-response group, else into the Response group. The sensitivity and specificity of the model were 0.722 and 0.724, respectively.

## 4 Discussion/conclusion

The presence of PVTT is classified as advanced stage HCC in the AASLD/BCLC staging system, and hepatectomy has not been recommended for more than a decade (Forner et al., 2018). However, recently many studies have reported that patients with PVTT can benefit from surgical resection compared with other treatments (Tanaka et al., 1996; Poon et al., 2003; Kokudo et al., 2016). Even so, the survival of patients with PVTT is dissatisfactory. The current study revealed that neoadjuvant FOLFOX-HAIC can prolong the survival of patients with PVTT who underwent hepatectomy. In addition, neoadjuvant FOLFOX-HAIC did not cause worse short-term outcomes, such as longer operation time, more operative blood loss, more postoperative complications and more 90-day mortality. In contrast, postoperative hospital stays were shorter in the HAIC-Surgery group. Tumor shrink after neoadjuvant HAIC may demonstrate the shorter postoperative hospital stays in the HAIC-Surgery group. The smaller the tumor, the more liver remains, the faster the recovery.

A large-scale cohort study based on the data available from Japanese nationwide survey showed that the median survival time for the liver resection (LR) group was 1.77 years longer than that for the non-LR group [2.87 years (95% CI, 2.60–3.37) vs. 1.10 years (95% CI, 1.03–1.17); *p* < 0.001] in HCC patients with PVTT. However, the survival of patients in the LR group was still poor, and the survival rates at 1, 3, and 5 years after diagnosis were 74.8%, 49.1%, and 39.1% for the LR group (Kokudo et al., 2016). In current study, the survival rates for Surgery group at 1, 3, and 5 years were 84.6%, 47.6% and 37.2%, which were similar to those in Kokudo’s study (Kokudo et al., 2016). Neoadjuvant therapy has been advocated to improve the postoperative prognoses of these patients. Wei and others reported that neoadjuvant radiotherapy provided significantly better post-operative survival outcomes than surgery alone for patients with resectable HCC and PVTT (Wei et al., 2019).

In recent studies, FOLFOX-HAIC presented promising therapeutic effects for advanced HCC with PVTT (He et al., 2017; He et al., 2018; He et al., 2019). He’s study demonstrated that FOLFOX-HAIC yielded a higher objective response rate and fewer adverse events than TACE for large HCC (He et al., 2017). The tumor of patients with PVTT was usually large and the average tumor size was 9.35 cm in this study, so HAIC is more suitable for patients with PVTT than TACE. The ORR for FOLFOX-HAIC is high (60.0%) in the current study, which is similar to that (52.6%) in He’s study. In addition, oxaliplatin-based HAIC might be more effective than cisplatin-based HAIC against HCC because of the advantage of the mechanism of cytotoxic action and the pharmacokinetics of HAIC (Dzodic et al., 2004; Bruno et al., 2017). Those demonstrated above may be the reason why neoadjuvant FOLFOX-HAIC can benefit HCC patients with PVTT receiving hepatectomy.

In the subgroup analysis, we found that the survival of patients responding to neoadjuvant FOLFOX-HAIC was significantly longer than that of non-responders. Interestingly, there was no significant difference in survival between the Non-response group and the Surgery group. The survival benefit of neoadjuvant FOLFOX-HAIC was limited to the responders. In addition, the times of HAIC procedures did not influence the survival of patients in the HAIC-surgery group. The survival of patients in the HAIC-Surgery group depended on the response to neoadjuvant FOLFOX-HAIC rather than times of HAIC. Therefore, it is vital to filter proper patients to accept neoadjuvant FOLFOX-HAIC, aiming to reduce unnecessary therapy for patients who cannot benefit from neoadjuvant FOLFOX-HAIC. We developed a logistic model to predict the response to neoadjuvant FOLFOX-HAIC. We found that baseline serum AFP and CRP levels were independent predictors of response. Both AFP and CRP are well known prognostic factors in HCC and have been incorporated in different prognostic models (Sieghart et al., 2013; Hucke et al., 2014; Miyaki et al., 2015; Mori et al., 2015). We included AFP and CRP in the logistic model. The model performed good predictive value with an AUC of 0.756 (95% IC, 0.627–0.868), which may be helpful for clinicians in determining proper treatment strategies for different patients. In myeloma studies, CRP enhanced cell proliferation and prevented chemotherapy-induced apoptosis (Yang et al., 2007), which may demonstrate that patients with high levels of CRP had a worse response to FOLFOX-HAIC. Interestingly, two recent Japanese studies developed and validated the CRAFITY score, consisting only of AFP and CRP, which predicts the response to immunotherapy and/or targeted therapy in HCC patients (Hatanaka et al., 2022; Scheiner et al., 2022).

In current study, neoadjuvant FOLFOX-HAIC significantly prolonged the survival of patients with Vp3/4. In patients with Vp1/2, the survival benefit of neoadjuvant FOLFOX-HAIC was not statistically significant. The therapeutic effect of neoadjuvant FOLFOX-HAIC was more prominent in patients with Vp3/4 than with Vp1/2. Kokudo’s study reported that LR is associated with a longer survival outcome than non-surgical treatment in patients with PVTT except Vp4 (Kokudo et al., 2016). Considering that a complete resection is extremely difficult in patients with advanced PVTT (Vp3/4), neoadjuvant therapy is required to shrink the PVTT. However, in patients with Vp1/2, complete resection is available without neoadjuvant FOLFOX-HAIC. As long as the PVTT located in main trunk/contralateral branch or first order branch, neoadjuvant FOLFOX-HAIC is necessary when liver resection is possible.

Since this investigation was a single-center and retrospective study, the unintentional selection bias is inevitable and the number of cases was small. Therefore, it is necessary to investigate this treatment strategy at multiple centers via a prospective study to confirm our findings.

In conclusion, a good prognosis could be obtained by performing neoadjuvant FOLFOX-HAIC in patients with PVTT who underwent hepatectomy. The survival benefit was limited to patients who responded to neoadjuvant FOLFOX-HAIC and the therapeutic effect of neoadjuvant HAIC was more prominent in patients with Vp3/4 than with Vp1/2. A logistic model that could predict the response to neoadjuvant FOLFOX-HAIC was developed and performed well.

## Data Availability

The original contributions presented in the study are included in the article/Supplementary Material, further inquiries can be directed to the corresponding authors.

## Supplementary material

SUPPLEMENTARY FIGURE S1 The magnetic resonance imaging of PVTT [red arrows, **(A)**]; Kaplan–Meier curves of overall survival between the Surgery group and the HAIC-Surgery group in patients including those who were unable to undergo hepatectomy after HAIC **(B)**; Kaplan–Meier curves of overall survival **(C)** and HCC recurrence **(D)** between the HAIC< 4 group and the HAIC >4 group.

## Abbreviations

AFP, alpha fetoprotein; CI, confidence interval; CR, complete response; CRP, C-reactive protein; FOLFOX, oxaliplatin, fluorouracil, and folinic acid; HAIC, neoadjuvant hepatic arterial infusion chemotherapy; HCC, hepatocellular carcinoma; MRI, Magnetic resonance imaging; MST, median survival time; ORR, overall response rate; OS, overall survival; PD, progressive disease; PR, partial response; PVTT, portal vein tumor thrombus; RFS, recurrence-free survival; SD, stable disease; TACE, transarterial chemoembolization.
