# Supplementary material for: Survival benefit of neoadjuvant hepatic arterial infusion chemotherapy followed by hepatectomy for hepatocellular carcinoma with portal vein tumor thrombus
Source: Front Pharmacol. 2023 Sep 19;14:1223632. doi: 10.3389/fphar.2023.1223632 (PMC10549930; doi:10.3389/fphar.2023.1223632)
Supplement: Supplementary file 1 [file Table1.docx]

**Table S1: Treatment pattern of patients with initial recurrence after liver resection**

|  | **Entire cohort** | | |  | **PSM cohort** | | |
| --- | --- | --- | --- | --- | --- | --- | --- |
|  | **Surgery group** | **HAIC-Surgery group** | **P-value** |  | **Surgery group** | **HAIC-Surgery group** | **P-value** |
|  | **(n=26)** | **(n=32)** |  |  | **(n=24)** | **(n=17)** |  |
| Radical treatment |  |  | 0.892 |  |  |  | 0.450 |
| Re-resection | 0 (0) | 1 (3.1) |  |  | 0 (0) | 1 (5.9) |  |
| Ablation | 6 (23.1) | 8 (25.0) |  |  | 6 (25.0) | 6 (35.3) |  |
| Palliative treatment |  |  |  |  |  |  |  |
| TACE/HAIC | 17 (65.4) | 16 (50.0) |  |  | 16 (66.7) | 4 (23.5) |  |
| TKI | 3 (11.5) | 5 (15.6) |  |  | 2 (8.3) | 4 (23.5) |  |
| TKI+ICI | 0 (0) | 2 (6.3) |  |  | 0 (0) | 2 (11.8) |  |

HAIC hepatic arterial infusion chemotherapy, ICI immune checkpoint inhibitor, TACE transarterial chemoembolization, TKI tyrosine kinase inhibitors.
