# Supplementary figures and images for: Survival benefit of neoadjuvant hepatic arterial infusion chemotherapy followed by hepatectomy for hepatocellular carcinoma with portal vein tumor thrombus
Source: Front Pharmacol. 2023 Sep 19;14:1223632. doi: 10.3389/fphar.2023.1223632 (PMC10549930; doi:10.3389/fphar.2023.1223632)

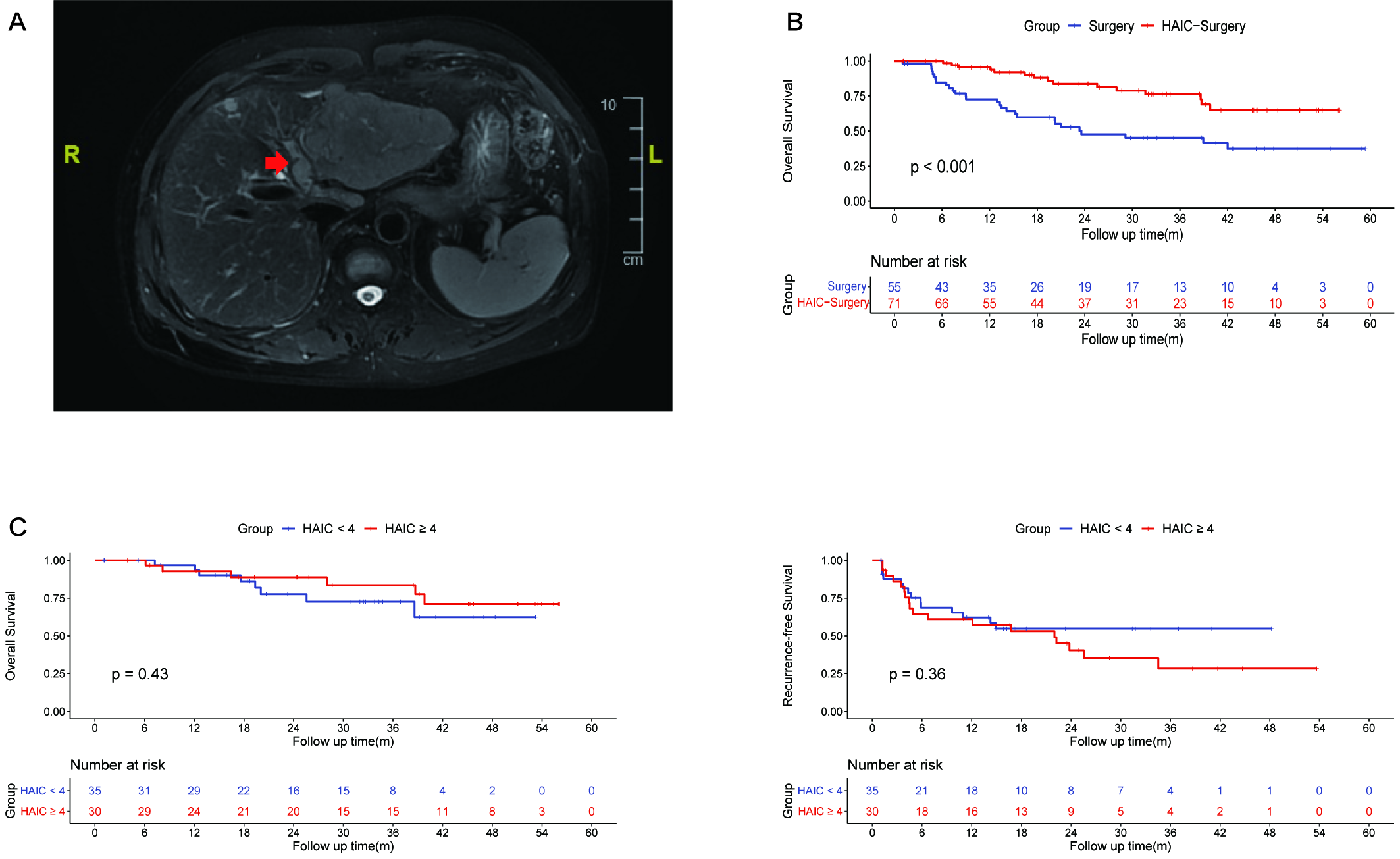

Supplement: Supplementary file 2 [file Image1.tif]
